# Public Narratives About Food Security Policies for Remote Aboriginal and Torres Strait Islander Communities

**DOI:** 10.1002/hpja.70102

**Published:** 2025-09-15

**Authors:** Cherie Russell, Lisa‐Maree Herron, Megan Ferguson, Caroline Deen, Emma Stubbs, Bronwyn Fredericks, Kani Thompson, Julie Brimblecombe, Amanda Lee, Ellie Chan, Emma Tonkin, Sue Kleve, Emma Chappell, Katherine Cullerton

**Affiliations:** ^1^ Institute for Health Transformation, Global Centre for Preventive Health and Nutrition, School of Medicine, Faculty of Health Deakin University Geelong Victoria Australia; ^2^ School of Public Health University of Queensland Herston Queensland Australia; ^3^ Wellbeing and Preventable Chronic Disease Division, Menzies School of Health Research Charles Darwin University Northwest Territories Australia; ^4^ Apunipima Cape York Health Council Bungalow Queensland Australia; ^5^ Sydney School of Health Sciences University of Sydney Lismore New South Wales Australia; ^6^ Central Australian Aboriginal Congress Alice Springs Northern Territory Australia; ^7^ Office of the Pro‐Vice Chancellor (Indigenous Engagement) University of Queensland St. Lucia Queensland Australia; ^8^ Department of Nutrition, Dietetics and Food, Faculty of Medicine Nursing and Health Sciences Monash University Clayton Victoria Australia

**Keywords:** Aboriginal and Torres Strait Islander, food and nutrition policy, food security, intercept method, narratives

## Abstract

**Issue Addressed:**

Despite ongoing efforts, meaningful policy action to improve food security in remote Aboriginal and Torres Strait Islander communities in Australia remains limited. Compelling, values‐based messaging may help drive change. This study explored public support for potential policies and the beliefs underpinning support or opposition, to inform future messaging on this issue.

**Methods:**

We used street‐intercepts to collect quantitative and qualitative data on community‐identified policy actions. Narrative analysis identified underlying beliefs and structural elements of differing narratives.

**Results:**

There was strong public support for government action, particularly for community‐driven solutions such as community farming and healthy food subsidies. Policies such as increasing welfare payments received less support. Two key narratives were identified: one framing food security as a human right and government responsibility, and the other emphasising community self‐determination and self‐sufficiency.

**Conclusions:**

While support for improved food security in remote communities was high, the preferred options and rationales varied. The narratives used to explain support or opposition offer insights into beliefs and attitudes that may be salient to the wider population.

**So What?:**

Narrative‐informed messaging could enhance public and policymaker support for community‐led strategies to improve food security.

## 1 Introduction

In the context of policymaking, how evidence and issues are communicated can influence public attitudes, opinions and support for an issue [1]. Public support, or a perceived lack of it, is a key factor influencing politicians' support for a particular policy [2]. One way to build support among both the public and policymakers is through the use of well‐crafted, strategic messages [2]. In particular, narratives have been shown to increase the resonance and impact of a policy message [3]. Effective narratives can engage audiences, enhance understanding, build support for an issue and motivate action [3, 4].

Impactful messaging is essential to drive action on complex public health issues such as food insecurity—an escalating issue in many countries, including Australia. Food security exists when all people at all times have ‘physical, social and economic access to food, which is safe and consumed in sufficient quantity and quality to meet their dietary needs and food preferences’ [5]. Prolonged food insecurity is associated with health inequity and outcomes such as increased rates of mental health conditions, heart disease, and premature mortality [6]. For children, it can hinder physical, social and emotional development and outcomes [7].

Aboriginal and Torres Strait Islander peoples are the Indigenous and first peoples of Australia. Before colonisation, Aboriginal and Torres Strait Islander peoples maintained food security through thousands of years of specialised knowledge of Country and its food systems [8]. Colonisation disrupted access to traditional lands, cultural knowledge, and food systems, with adverse consequences for food security and nutritional health [9, 10]. These disruptions, compounded by ongoing structural inequities and policies that perpetuate inadequate living conditions (inadequate housing, limited infrastructure, poor food storage and preparation facilities; restricted access to transportation), inadequate employment opportunities, and low incomes [11] underpin continuing high rates of food insecurity. Increasing numbers of Aboriginal and Torres Strait Islander families in urban and regional areas are affected [12], while in remote communities where food is consistently more expensive than non‐remote areas, many households face ongoing barriers to accessing sufficient and quality food [13, 14]. The National Aboriginal and Torres Strait Islander Health Survey (2022–23) found that half (51%) of Aboriginal and Torres Strait Islander households in remote areas reported food insecurity (defined as experiencing food insecurity due to a lack of money for food at some time in the previous 12 months) [15].

Governments at all levels (federal, state/territory, and local) have a vital role in addressing food security for all Australians [16]. In remote Aboriginal and Torres Strait Islander communities, local initiatives like healthy food price discounts in community stores are valuable, but broader structural changes, such as improved roads, require state and national action. Research suggests that while local solutions are important, some solutions need government action in genuine partnership with Aboriginal and Torres Strait Islander communities to ensure they maintain the right to control decisions affecting their wellbeing [17, 18]. However, to date, limited meaningful policy action from the government, with or without community support, has been implemented [19]. There are many factors contributing to the lack of policy investment, including the issue not resonating with policymakers [2].

To increase policymakers' interest in these policies, Chong and Druckman [1] have argued it is essential to develop effective messages that resonate with the audience's social reality and their political ideology. This requires a deep understanding of the target audience's shared societal values, commonly used language and narratives that shape public attitudes towards these policies.

While recent research has examined public opinion on broader public health nutrition policies in Australia [20], there has been limited exploration of public attitudes towards government action to improve food security in remote Aboriginal and Torres Strait Islander communities. This study aimed to address that gap by exploring public attitudes and levels of support for policies identified by communities themselves, and by critically analysing the underlying narratives that inform these attitudes.

## 2 Methods

To answer our research questions: (a) the extent of public support for potential policies to improve food security, and (b) the beliefs underpinning support or a neutrality towards these policies that may inform the development of effective future messaging strategies, we used a mixed methods approach. This involved a street‐intercept interview tool that collected both quantitative data through closed questions and qualitative data through open‐ended questions.

### 2.1 Research Governance

This study was part of a Remote Food Security Project, developed in response to calls to action from Aboriginal Community Controlled Health Organisations (ACCHO) including Apunipima Cape York Health Council [21] and Central Australian Aboriginal Congress [22]. That broader project, which aimed to identify community‐led solutions to food security in remote communities, was co‐designed by Aboriginal and Torres Strait Islander and non‐Indigenous staff and nutrition, food security and policy academics [17]. Guided by local Community Advisory Groups in remote communities in Cape York and Central Australia, the design took a strengths‐based approach, supporting and recognising human rights and social justice, along with a broader empowerment agenda. Within this governance structure, a working group comprising ACCHO and academic project team members and public health nutrition experts oversaw the design and implementation of the street‐intercept interview study. It was approved by the Research Governance Committee of Apunipima Cape York Health Council and the Central Australian Aboriginal Congress Board.

### 2.2 Data Collection

Data were collected through street‐intercept interviews. This method enables access to a broader range of individuals across demographic categories, and higher response rates and less bias than online, mail, and telephone surveys [23, 24]. Street‐intercept interviews were conducted by five trained interviewers between November 2022 and February 2023, in urban and regional locations in the Australian states of Victoria and Queensland. Interviewers approached potential participants (members of the public who were sitting or standing alone and appeared to be adults with capacity to engage in the survey) in public locations, such as parks and shopping precincts, and invited them to complete an interview. To be eligible, individuals had to be over 18 years of age, speak English, and be eligible to vote in Australia. They were given a plain language statement and signed a consent form agreeing to participate and be audio recorded prior to the commencement of the interview. Recruitment continued until data saturation was reached (i.e., the interviews offered no further new insights). Interview duration ranged from 4.5 to 20 min, with an average of 7.5 min.

Participants were asked their level of agreement (using a scale from 1 being ‘*strongly disagree*’ to 5 being ‘*strongly agree*’) for each of 10 different policy options (Box 1). The policy options were community‐generated, derived from earlier research in the Remote Food Security Project, including a photovoice study exploring solutions to food insecurity from the perspective of caregivers of children [14], and workshops with community leaders to prioritise potential strategies to improve food security [17]. These strategies were refined by the study's working group to develop the 10 policy options included in the street‐intercept interviews.

BOX 1 **Policy Options.**

- Support the development of community‐owned farming/gardening operations which provide food, local employment, skill development, and secure jobs for people living in remote Aboriginal and Torres Strait Islander communities;
- Improve the maintenance of public housing, including kitchen items e.g., stoves and items needed for safely preparing and storing food, for people living in remote Aboriginal and Torres Strait Islander communities;
- Improve the quality of roads between remote communities and major towns to improve access for community members and for food delivery;
- Increase public transport between remote communities and major towns to improve shopping options for remote community residents;
- Provide subsidies for healthy foods, making them more affordable for people living in remote Aboriginal and Torres Strait Islander communities;
- Subsidise electricity, making it more affordable for people living in remote Aboriginal and Torres Strait Islander communities;
- Increase the regularity of current welfare payments for people living in remote Aboriginal and Torres Strait Islander communities so recipients can receive payments more frequently;
- Increase support for job seekers and employment opportunities for local people in remote Aboriginal and Torres Strait Islander communities;
- Increase the availability of public housing and simplify the process of applying for public housing for people living in remote Aboriginal and Torres Strait Islander communities;
- Increase the total amount of welfare payments people receive fortnightly for people living in remote Aboriginal and Torres Strait Islander communities.

To collect qualitative data, interviewers focused on the policy options that elicited strong agreement or disagreement and asked participants to explain the reasons for their beliefs. We also asked participants if they had additional comments or policy options they would like to add. We recorded demographic data for each participant, including gender, age range, highest level of education, postcode, and political party they previously voted for.

### 2.3 Data Analysis

#### 2.3.1 Quantitative Analysis

Demographic characteristics of participants were transferred from survey forms to a Microsoft Excel spreadsheet to calculate descriptive statistics. These included mean agreement scores for each demographic category and policy option, ranging from a minimum of 1 to a maximum of 5 (highest agreement). Political views were classified as ‘progressive’ if the participant identified as previously voting for the Greens Party, Australian Labor Party or Socialist Party, and ‘conservative’ if they voted for the National Party, Australian Liberal Party, One Nation or The Freedom Party. Undecided or ‘swing’ voters, and individuals who said they voted for no party or for independent candidates, were classified as ‘other/none’. Participants' postcodes were used to determine geographical classifications, using the Accessibility Remoteness Index of Australia Plus [25], and socioeconomic status (SES), using the Socio‐Economic Indexes for Areas Index of Relative Socio‐economic Disadvantage [26]. As there were few participants whose residential postcode was classified as ‘rural’, they were grouped as ‘regional/rural’.

#### 2.3.2 Qualitative Analysis

Interview audio files were de‐identified and transcribed. After familiarisation with the data, we developed an initial coding schema in NVivo qualitative analysis software (QSR International, Version 12) based on the Qualitative Narrative Policy Framework (QNPF) [27]. As data analysis progressed, this was inductively modified to reflect the aims of the research and the themes identified. The QNPF, used in previous similar analyses [20], can be used to systematically understand the role of narratives and beliefs in policy support and processes by demonstrating the underlying structural elements to differing narratives [27]. Narrative elements, as described by Cullerton et al. [20], are shown in Table 1.

**TABLE 1:** Narrative elements based on the qualitative narrative policy framework [20, 27].

Element	Definition
Setting	The setting is the space where the action of the story takes place over time
Characters	Actors are often seen or described as ‘victims’ that are harmed by the problem, ‘villains’ that intentionally or unintentionally cause the harm and ‘heroes’ that provide or promise relief from the harm
Moral of the story	The policy solution promoted by a policy narrative
Plot	Plots explain the connections between the elements of the narrative
Belief systems	Ideologies and beliefs based on what individuals perceive as their reality (determined using which party they vote for)

During initial coding, the first coder removed transcript content identified as racist; this was defined as statements that demeaned, stereotyped, or expressed prejudice or discrimination towards Aboriginal or Torres Strait Islander individuals, communities, or the population as a whole. This approach aligned with the study's aim to identify positive or neutral narratives to inform future messaging. While such views are part of the broader discourse, they do not contribute to the development of effective messaging strategies. This is because racist narratives risk alienating broader audiences, deepening social divisions, and do not offer constructive frames that can be used to persuade or unify. Moreover, repeating or engaging with these views can inadvertently reinforce them through increased familiarity [1]. Ethically and methodologically, our focus was on identifying beliefs and narratives that could assist in unifying, persuading, and promoting positive outcomes.

Following initial coding, a second coder double coded all transcripts. To ensure meaning was considered from the perspective of an Aboriginal and/or Torres Strait Islander person and to improve research rigour, 10% of the transcripts were double coded by Aboriginal researchers (ES and CD deidentified for review). Findings were discussed and confirmed by all authors.

## 3 Results

### 3.1 Quantitative Results

In total, 85 people participated in the street intercept interviews, a response rate of 51%. Participant demographic variables were reasonably evenly distributed for gender and age but regional/rural participants and conservative voters were underrepresented (Table 2). One participant declined audio recording, and one audio file was corrupted, so 83 recorded interviews were analysed.

**TABLE 2:** Demographic characteristics (*n* = 85) and mean agreement scores for each policy by location and political views.

	*n* (%)	A	B	C	D	E	F	G	H	I	J
Mean agreement		4.59	4.36	4.42	4.32	4.52	4.24	3.74	4.45	4.23	3.93
Gender											
Male	39 (46%)	4.38	4.21	4.38	4.18	4.51	4.1	3.62	4.39	4.15	3.83
Female	45 (53%)	4.76	4.49	4.49	4.58	4.52	4.34	3.84	4.49	4.29	4.04
Other	1 (1%)	5	5	3	4	5	5	4	5	5	3
Age range (years)											
18–24	17 (20%)	4.82	4.47	4.47	4.76	4.76	4.59	4.12	4.82	4.53	4
24–44	29 (34%)	4.45	4.48	4.55	4.38	4.53	4.4	3.72	4.45	4.38	4.16
45–64	22 (26%)	4.59	4.27	4.45	4.45	4.36	4.05	3.68	4.36	4.05	3.82
65+	17 (20%)	4.59	4.18	4.12	3.94	4.47	3.88	3.47	4.19	3.94	3.65
Education											
None	1 (1%)	5	5	5	5	5	5	5	5	5	5
High school	25 (29%)	4.48	4.52	4.2	4.44	4.56	4.12	3.76	4.28	4.32	3.88
Diploma	4 (5%)	5	5	5	5	5	5	3.75	5	4.75	4.25
Undergraduate	18 (21%)	4.83	4.44	4.56	4.44	4.56	4.28	4.39	4.56	4.56	4.33
Post‐graduate	37 (44%)	4.49	4.14	4.43	4.24	4.42	4.2	3.38	4.46	3.95	3.72
Political views											
Progressive	47 (55%)	4.66	4.49	4.49	4.43	4.62	4.47	4.06	4.55	4.53	4.3
Conservative	14 (17%)	4.64	4.29	4.29	4.21	4.43	4.29	3.36	4.31	3.64	3.39
None/other	24 (28%)	4.42	4.17	4.38	4.42	4.4	3.77	3.33	4.33	4	3.54
Location											
Urban	70 (82%)	4.59	4.46	4.46	4.41	4.52	4.38	3.77	4.51	4.24	4.05
Regional/rural	15 (18%)	4.6	3.93	4.27	4.27	4.53	3.6	3.6	4.2	4.2	3.4
Socioeconomic status											
Low (1–4)	15 (18%)	4.67	4.27	4.33	4.4	4.73	4.2	3.53	4.43	4.4	3.73
Medium (5, 6)	18 (22%)	4.32	4.21	4.53	4.37	4.79	4.37	3.89	4.21	4.16	4.08
High (7–10)	51 (60%)	4.67	4.45	4.41	4.39	4.36	4.21	3.75	4.55	4.22	3.94

*Note:* A. Support the development of community owned farming/gardening operations which provide food, local employment, skill development, and secure jobs for people living in remote Aboriginal and Torres Strait Islander communities. B. Improve the maintenance of public housing, including kitchen items e.g., stoves and items needed for safely preparing and storing food, for people living in remote Aboriginal and Torres Strait Islander communities. C. Improve the quality of roads between remote communities and major towns to improve access for community members and for food delivery. D. Increase public transport between remote communities and major towns to improve shopping options for remote community residents. E. Provide subsidies for healthy foods, making them more affordable for people living in remote Aboriginal and Torres Strait Islander communities. F. Subsidise electricity, making it more affordable for people living in remote Aboriginal and Torres Strait Islander communities. G. Increase the regularity of current welfare payments for people living in remote Aboriginal and Torres Strait Islander communities so recipients can receive payments more frequently. H. Increase support for job seekers and employment opportunities for local people in remote Aboriginal and Torres Strait Islander communities. I. Increase the availability of public housing and simplify the process of applying for public housing for people living in remote Aboriginal and Torres Strait Islander communities. J. Increase the total amount of welfare payments people receive fortnightly for people living in remote Aboriginal and Torres Strait Islander communities.

Of the 83 participants with complete interview data, 79 (95%) agreed when asked whether the Australian Government should do more in partnership with communities to improve access to healthy food for people living in remote Aboriginal and Torres Strait Islander communities. Overall, public support was high for most policy options, with all but one receiving a mean agreement score above 3.9. The strongest support was for the development of community‐owned farming and gardening operations (mean agreement of 4.59), healthy food subsidies (4.52) and increased employment opportunities and support for job seekers (4.45). Policies that received lower levels of agreement included proposals to increase the frequency (3.74) or total amount (3.94) of welfare payments (Table 2).

### 3.2 Qualitative Results

The qualitative results provided insights as to why participants agreed or disagreed with the proposed policy actions and with government involvement generally. They highlighted the narratives participants used when discussing food security policies and the beliefs underlying these views. While participants generally agreed on the need for government action in collaboration with Aboriginal and Torres Strait Islander communities to improve food security, reasons for this support—and the nature of it—differed. Two key narratives related to policy to improve food security among Aboriginal and Torres Strait Islander peoples were identified: (i) an obligation of the Australian government to its citizens, and (ii) ‘supporting, not giving’: a preference for policy actions seen as enabling individual or community self‐determination or self‐sufficiency. These narratives, including their relevant settings, characters, morals and plots and rationales for solutions as described by participants, are discussed below. Please note, for Aboriginal and Torres Strait Islander readers, some quotes used to demonstrate results may be confronting.

#### 3.2.1 Ensuring Food Security Is a Government Responsibility

Several participants described action in collaboration with communities to improve food security as an important responsibility of Australian governments, with two dominant narratives offered.

Some described access to healthy food as a human right—with the implication that it should be assured by governments for all citizens.

It's a human rights thing. In other words, for all people, access to healthy, reliable food is a human rights issue. (Male, 45–64, Urban, Progressive voter)

Well, everyone deserves a right to have healthy food. (Female, 45–64, Urban, No political preference)

The second narrative was that government action was necessary to address inequities. Several participants described the inequitable impact of social determinants and specific barriers to accessible and affordable food in remote Aboriginal and Torres Strait Islander communities, necessitating government intervention to improve food security.

There's just basic needs that aren't being met, like secure housing, a liveable income, access to fresh food and health services—everything that you need to live a healthy life. (Female, 25–44, Urban, Progressive voter)

The moral of both sentiments in this narrative was that improving food security was primarily the responsibility of governments, but collaboration with community was crucial to ensure policy initiatives would be appropriate and culturally sensitive.

I think that the government needs to listen to [Aboriginal and Torres Strait Islander people] as well. They may be coming up with some very, very important points of how they have lived their life for all these years … whatever that is best for them. (Male, 45–64, Urban, No political preference)

Some participants emphasised the impacts of colonisation as rationale for government action to address food security in Aboriginal and Torres Strait Islander communities. This connection not only situated food insecurity within a broader context of systemic injustice but also framed government action as a necessary form of redress and reconciliation:

The government has responsibility over its citizens and particularly First Nations people [who] have been oppressed for a long time. (Male, 18–24, Rural, Progressive voter)

I don't know enough about the situation on the ground to be specific, but generally, historically, I think there hasn't been enough care taken of Aboriginal and Torres Strait Islander peoples. (Female, > 65, Urban, No political preference)

As such, government was characterised as both the ‘villain’—the cause of current food security problems—and the potential ‘hero’, with the power and responsibility to address the issue.

I think there's clearly a need to improve things, and I just think it's very much the government's and the taxpayer's responsibility to fund it. (Female, 18–24, Urban, Progressive voter)

Participants described government culpability in terms of poor resource allocation by state and federal governments, and emphasised the need for decentralised, community‐informed approaches that shift not only funding but also decision‐making closer to those impacted:

Until we can better distribute that wealth across the regions, the regions are just going to struggle so much. It's all centred in these capital cities, that's where the bureaucrats are, and they don't want it to change. (Female, 25–44, Urban, Progressive voter)

Although the interview questions were focused on policy to improve food security in remote Aboriginal and Torres Strait Islander communities, in their responses many participants broadened their narrative or frame of reference to all Australians. They were supportive of the policy options to subsidise the cost of healthy foods and electricity and increase welfare payments for people living in remote Aboriginal and Torres Strait Islander communities but also wanted to express their view that many Australians were in need of financial assistance and should be the beneficiaries of government policy action:

If you are asking me about subsidies for healthy foods … it should be for everyone. (Female, 25–44, Urban, No political preference)

Make [electricity] cheaper. I wish everything was cheaper for everyone. (Female, 25–44, Urban, Conservative voter)

#### 3.2.2 Self‐Sufficiency Versus Self‐Determination

A second narrative that underpinned support for a particular policy or government action in general was that policy was a mechanism to support either household or community ‘self‐sufficiency’. There were two clear and distinct ways participants framed their responses: as support for policy action to encourage individual or family *self‐sufficiency*, in place of ongoing government support; or as support for policies that enabled community *self‐determination*, as a right of Aboriginal and Torres Strait Islander peoples.

Participants using the ‘self‐sufficiency’ narrative supported policy actions that placed responsibility on Aboriginal and Torres Strait Islander people and communities for food production while showing limited support for policy actions they interpreted as ongoing assistance, such as increased welfare payments.

It teaches people. It's like buy a person a fish or teach a person to fish. You give them the skill, and then after time, hopefully, it becomes embedded. That's why I don't want subsidies because they're one‐off and a one‐way thing, where if you provide infrastructure like a hothouse or some way to grow foods locally, you teach… all that sort of stuff (Male, 45–64, urban, Progressive voter)

These participants presented the value of individual or community self‐sufficiency as rationale for strongly endorsing policy actions that they considered to be ‘supporting, not giving’ (Female, 18–24, Urban, Progressive voter), and not supporting policies like increasing welfare payments. For example:

…I strongly agreed because that's probably the future, it's not giving [people] more money, more resources. That's teaching them how to do it and how to be sustainable. (Female, 45–64, Regional, Conservative voter)

Rather than giving [job seekers] those payments, give them more … support to get employed and become independent rather than giving those payments … (Female, 25–44, Urban, No political preference)

Other participants used this narrative to support policy action aimed at increasing food security in remote Aboriginal and Torres Strait Islander communities by making it more affordable for households—including subsidies for healthy food and/or electricity and increased welfare payments. They viewed these measures as a government responsibility to facilitate greater self‐determination in communities, acknowledging inequities brought about by colonisation.

Given the background, the interaction between government and those communities … the idea would hopefully be empowering people to make their own decisions. (Female, 25–44, Urban, Progressive voter)

Many participants expressed a view that improving food security needs to be a joint effort of government and community—with governments responsible for developing policy and resourcing community‐identified solutions and initiatives.

I feel you want [policy action] to be culturally safe, right? … Maybe it might be a dual sort of thing, where it's a collaborative practice where recommendations are made from those communities and they're taken seriously on board and supported by the government, I guess. (Other gender, 18–24, Urban, Progressive voter)

In this way, both governments and communities were identified as heroes, with a joint power and responsibility to initiate and maintain change.

For some participants, a related moral was that any policy action to address food security needed to incorporate and promote Aboriginal and Torres Strait Islander cultures and traditional food practices. This moral grounds the narrative in cultural resilience and Indigenous knowledge as key assets:

They've managed to live in Australia for all these years knowing that Australia is more like a desert, and they survived … it'd be interesting to know how they become so resilient. And then we can incorporate that into policies, into what the government needs to do. (Male, 45–64, Urban, No political preference)

Further, many participants who supported policy solutions encouraging self‐sufficiency described successful policies as those that were pragmatic, practical or cost‐effective. Despite the strength of this theme throughout the interviews, specific examples were subjective, without a clear consensus on what would be a cost effective or pragmatic policy.

Only on a case by case. Only after a cost benefit analysis demonstrates that, yeah, you can do this in long term, that it'll be very beneficial and you're not throwing taxpayer money down the drain in a way. (Female, 45–65, Urban, Progressive voter)

#### 3.2.3 Other Policy Solutions

In response to the interviewers' prompts, some participants offered other policy solutions they felt could improve food security in remote communities. The most commonly proposed was education about ‘healthy eating’ to support individual behaviour change. For example:

There should also maybe be some education around healthy foods. … So I feel like education, even at a primary school to high school level, could be good as well. (Female, 18–24, Urban, No political preference)

However, another participant noted the limitations of education in the context of broader determinants of food security, especially in contexts where structural barriers, such as low incomes and limited employment opportunities as well as the high cost of healthy food, persist:

There's no point in educating people if it's then impossible to obtain [healthy food] anyway because it's too expensive. So, I think it needs to go hand in hand: lessen the costs, and then also tell people about it. (Female, 18–24, Regional, Progressive voter)

## 4 Discussion

The aim of this study was to explore support for and public attitudes towards Aboriginal and Torres Strait Islander community‐identified policies to improve food security in remote communities, and to analyse the underlying narratives shaping these attitudes. Most participants (95%) supported government collaboration with Aboriginal and Torres Strait Islander communities to improve food access and security in remote areas. This remarkably high level of support demonstrates the possible salience of this issue among the voting public in Australia and an opportunity to leverage it in advocacy aimed at policymakers.

Public support for each of the proposed policy options was overwhelmingly positive, with all but one receiving a mean agreement score above 3.9 out of 5. Establishing community‐owned farming and gardening operations was the most supported policy option. Community gardening is an oft‐cited solution, in both urban and rural areas, to address food insecurity [19, 28]. Community gardens have also been suggested as an aide to reduce food insecurity in a previous study with urban and regional Aboriginal and Torres Strait Islander Communities [12]. While community gardens represent a means of agency for communities to promote food security, nutrition, biodiversity and mitigating climate change, in isolation, community gardening does not address the broader, systemic drivers of food insecurity [19].

In contrast, the policy options with the least agreement were those related to fiscal policies designed to address systemic drivers, particularly increasing the regularity of welfare payments. In a previous study using street intercept interviews to determine public support for more general nutrition policies, fiscal strategies, such as taxes, were also the least popular among participants [20].

The youngest participants (18–24 years) tended to show the highest level of agreement across all policies, with agreement decreasing among older age groups. These findings align with previous research showing that younger individuals tend to hold more left‐leaning political views [29], with greater affinity for socialism, welfare policies, and government intervention [30]. Participants who voted for the Australian Greens or Australian Labor Party exhibited higher average support, particularly for policies related to welfare payments, compared to conservative party supporters. This pattern is consistent with the political parties' ideological foundations [31].

There was a dominant narrative that policy actions should be aimed at enabling ‘self‐determination’ of Aboriginal and Torres Strait Islander communities, evident in the qualitative responses of many participants. The Lowitja Institute, an Aboriginal and Torres Strait Islander community‐controlled health research institute, has argued that self‐determination is crucial to ensure ongoing social, cultural, and political determinants of health and wellbeing are prioritised [32]. It is important to note that self‐determination must include Aboriginal and Torres Strait Islander leadership in policymaking that impacts community [33], an imperative highlighted by several study participants, and in other work [34]. This is a significant departure from some earlier government decision‐making that occurred without the due diligence of listening to, consulting, and co‐designing with Communities, as observed in other public health research [35].

The other dominant narrative was the obligation of the Australian government to its citizens, both characterising food security as a human right, and highlighting a government responsibility to right structural wrongs and enable a ‘fair go’ for Aboriginal and Torres Strait Islander peoples. In this narrative, governments were generally seen as perpetrators of the problem, but also as the entity with the power to address it, i.e., the ‘heroes’.

Recent research examining public support for regulatory measures designed to improve the nutritional status of all Australians, such as sugar taxes and banning the advertising of unhealthy foods to children, showed a prevailing individualistic or libertarian attitude to food policy [20]. This attitude was also reflected in the additional policy solutions suggested by participants in this study, such as food and nutrition education to support individual behaviour change. The suggestion of individualistic solutions to food security may reflect the broader framing of Aboriginal and Torres Strait Islander nutrition and health issues in media reporting [36, 37] and in Australian politicians' media releases and public statements [38].

To the best of our knowledge, this is the only survey of public opinion on health policies for Aboriginal and Torres Strait Islander communities, and one of the few that examines public support for any policy impacting on Australia's First Peoples. The results demonstrate the complexities of public opinion, highlighting the need for more nuanced communication strategies and deeper engagement to foster understanding and support for future initiatives.

## 5 Strengths and Limitations

This study's approach and the policy options were codesigned in partnership with several Aboriginal and Torres Strait Islander researchers, organisations and communities, to ensure the policy options proposed were culturally appropriate and supported by the communities impacted by their implementation. They align with community‐driven solutions [14] and suggestions from Aboriginal and Torres Strait Islander peoples in remote communities [13], as well as findings from previous research and government inquiries [39, 40].

The study has several limitations. Interviews were conducted only in two states (Queensland and Victoria) hence, the results may not be representative of the beliefs of Australians generally. Participants from rural and remote areas and those with conservative political views were underrepresented, potentially introducing a progressive bias. However, given that a Labor government held power federally and in most states and territories at the time of data collection, participants' political preferences may still reflect broader public sentiment. We acknowledge that excluding racist views limits representation of the full spectrum of public sentiment. However, our goal was not to map all perspectives but to strategically identify messaging opportunities for policy advancement. This necessitated a focus on constructive and ethically sound narratives. As a qualitative study, we do not claim representativeness, and given this is the first Australian study exploring public salience of this policy issue, we are unable to assess changes in attitudes over time. Finally, our primary focus was to identify high‐level narrative themes and overarching insights across the interview dataset rather than conduct a granular, policy‐by‐policy analysis. We aimed to understand broader patterns in how participants conceptualise government involvement and community collaboration in addressing food security. While a more detailed examination of the rationales behind support for individual policies could offer nuanced recommendations for government partnerships, we suggest this as a direction for future research.

## 6 Conclusion

This study found high levels of public support for policies to improve food security in remote Aboriginal and Torres Strait Islander communities, though preferred options and rationales for support varied. The narratives participants used to explain why they did or did not support particular policies provide insights into beliefs and attitudes that may be salient to the wider population. However, as rural communities and conservative voters were under‐represented in this study, further research is needed to better understand their perspectives. This would help determine whether and how narratives of fairness, human rights, community co‐design, and self‐determination can be used in policy messaging among these groups to gain political traction. In addition, further research could be undertaken to examine the rationales behind support for individual policies; this could yield nuanced recommendations for government partnerships.

## Ethics Statement

Ethics approval was granted by The University of Queensland International Human Research Ethics Committee (2020/HE000636) and the Central Australian Human Research Ethics Committee (CA‐20‐3701).

## Conflicts of Interest

The authors declare no conflicts of interest.

## Data Availability

The data that support the findings of this study are available from the corresponding author upon reasonable request.
